# Modeling Fretting Wear Resistance and Shakedown of Metallic Materials with Graded Nanostructured Surfaces

**DOI:** 10.3390/nano13101584

**Published:** 2023-05-09

**Authors:** Ting Yang, T. A. Venkatesh, Ming Dao

**Affiliations:** 1 Department of Materials Science and Engineering, Massachusetts Institute of Technology, Cambridge, MA 02139, USA; 2 Department of Materials Science and Chemical Engineering, Stony Brook University, Stony Brook, NY 11794, USA

**Keywords:** fretting, frictional sliding, graded nanostructured surfaces, shakedown, finite element

## Abstract

In applications involving fretting wear damage, surfaces with high yield strength and wear resistance are required. In this study, the mechanical responses of materials with graded nanostructured surfaces during fretting sliding are investigated and compared to homogeneous materials through a systematic computational study. A three-dimensional finite element model is developed to characterize the fretting sliding characteristics and shakedown behavior with varying degrees of contact friction and gradient layer thicknesses. Results obtained using a representative model material (i.e., 304 stainless steel) demonstrate that metallic materials with a graded nanostructured surface could exhibit a more than 80% reduction in plastically deformed surface areas and volumes, resulting in superior fretting damage resistance in comparison to homogeneous coarse-grained metals. In particular, a graded nanostructured material can exhibit elastic or plastic shakedown, depending on the contact friction coefficient. Optimal fretting resistance can be achieved for the graded nanostructured material by decreasing the friction coefficient (e.g., from 0.6 to 0.4 in 304 stainless steel), resulting in an elastic shakedown behavior, where the plastically deformed volume and area exhibit zero increment in the accumulated plastic strain during further sliding. These findings in the graded nanostructured materials using 304 stainless steel as a model system can be further tailored for engineering optimal fretting damage resistance.

## 1. Introduction

In several engineering structures, such as bolted or riveted connections, shaft couplings, and turbine blade–hub assemblies, cyclic loading can often lead to fretting damage in the structural components. This type of damage occurs at or near the contact surface due to fretting-induced plastic deformation at the surface and/or underneath the surface, which can significantly reduce the service life of the component materials. For example, in pressurized water reactors, at the regions where the spacers are in contact with the exterior of the fuel rods (i.e., the surface of the cladding that surrounds the radioactive fuel rod core), vibrational loads caused by turbulence in the water induce fretting damage, which can lead to cracking and deterioration in the cladding layer, resulting in potentially serious radioactive leaks. Therefore, it is extremely important that the fretting resistance of such materials be enhanced to improve the service life in these applications. 

Recent experimental studies have shown that various surface modification methods, based on mechanical [1], thermal [2], and electrochemical processes [3,4,5], can be used to enhance surface properties. Amongst several surface modification methods, the surface mechanical attrition treatment (SMAT) [1] method has emerged as a versatile technique that can help engineer a range of graded nanostructured (GNS) metallic materials including steels [6,7], copper [7], titanium [8], and alloys [9]. GNS metallic materials, such as graded nanostructured steels [10,11], gradient nanograined copper [12,13,14], and alloys [15], exhibit remarkable fatigue properties, even after undergoing significant high-cycle mechanical loading. In addition, GNS materials have also demonstrated superior resistance to friction [16,17,18,19,20], wear [17,18,19,20], and corrosion [21,22,23]. These properties make them ideal candidates for use in applications that require high durability and resistance to mechanical stresses. These GNS materials have a gradient in their internal microstructure, including grain size and twin or lamellar thickness that extends from the surface to the interior over a length scale ranging from several nanometers to millimeters. During the SMAT process, several millimeter-sized spherical steel shots are accelerated to high speeds by powerful ultrasonic or other energy-transfer modes and are made to impact the sample’s surface [7], which induces severe plastic deformation in the surface layers [24,25]. The coarse-grained structure in the surface layer is transformed into a nanograined structure without a change in its chemical composition due to the plastic deformation in the surface layer caused by the impact of the steel shots [26,27]. Depending on the SMAT conditions, a range of gradients in the distribution of plastic strain from the top surface to the interior can be generated. This gradient in the plastic strain distribution determines the degree of grain refining produced by the SMAT process in the substrate material. The GNS materials produced by the SMAT process can exhibit a graded microstructure with grain sizes in the order of nanometers near the top surface where there is maximum plastic strain accumulation [28,29]. GNS materials have been shown to exhibit superior sliding and fretting wear resistance [30,31], which is significantly affected by the contact friction conditions. It is speculated that their fretting characteristics may be enhanced relative to the untreated materials that exhibit a homogeneous, uniform coarse-grained structure. However, a systematic study that assesses the response of GNS materials that exhibit plasticity gradients to fretting conditions is not yet available.

Both analytical modeling and finite element modeling have been invoked to understand the mechanical response of substrate materials and the conditions of damage accumulation observed in experiments under several contact loading conditions. For example, an analysis of the sliding contact between an elastic homogenous half-space and a rigid circular sphere [32] or a rigid cylinder [33] was performed using analytical approaches, while finite element analysis and various analytical techniques have been utilized to investigate the elastic–plastic analysis of sliding and rolling contact [34,35,36,37]. Shakedown behavior under contact loading has also been extensively studied [38,39,40,41]. The phenomenon known as *elastic shakedown* occurs when a material deforms plastically on initial contact, but after a certain amount of fretting contact, the material responds with solely elastic behavior (i.e., no additional plastic deformation occurs). When the steady-state stress–strain response of the substrate material under contact loading is represented by a closed elastic–plastic loop, and there is no net change in the plastic strain (i.e., plastic strain tensor $\varepsilon^{p}\left(x,y,z\right)$ remains the same after each full fretting cycle) despite the increasing (accumulated) equivalent plastic strain (${\overset{-}{\varepsilon }}^{p}\left(x,y,z\right)$ (a scalar defined as ${\overset{-}{\varepsilon }}^{p}=\int_{0}^{t}\frac{2}{3}{\dot{\varepsilon }}^{p}:{\dot{\varepsilon }}^{p}dt$) with respect to the increasing number of fretting cycles, this phenomenon is referred to as *plastic shakedown* behavior in the literature [42,43]. For a more intuitive and practical definition, here in this study, we define *plastic shakedown* behavior to be the case when there is no increase in plastically deformed surface area and volume (where ${\overset{-}{\varepsilon }}^{p}\left(x,y,z\right)>$ 0) with an increasing number of fretting cycles, but the change in equivalent plastic strain—$∆{\overset{-}{\varepsilon }}^{p}\left(x,y,z\right)$—is not zero everywhere within the plastically deformed volume. Note that when $∆{\overset{-}{\varepsilon }}^{p}\left(x,y,z\right)$ is zero everywhere in the structure, the traditionally defined *elastic shakedown* is reached. Hereafter, we will use the updated definition of *plastic shakedown* unless otherwise noted. This allows us to include cases that fall between the traditionally defined *elastic shakedown* and *plastic shakedown* behaviors when the substrate material is not perfectly plastic.

While several studies have examined the mechanical response of homogeneous substrate materials to contact loading conditions, relatively fewer studies have focused on the contact behavior of materials with a layered microstructure. For example, the effects of a bi-layered microstructure in the substrate material on applied loads during repeated sliding contact have been investigated [42,44]. However, the sliding analysis was limited to two cycles of loading only. Furthermore, three-dimensional frictional sliding analysis that differentiates the contact behavior of graded materials from that of homogeneous (ungraded) materials is also limited [20]. Hence, the objectives of the present study are:

(1) To develop a three-dimensional finite element model to characterize the sliding contact behavior and the shakedown response of materials with graded nanostructures;
(2) To understand the relationships between contact load, contact geometry, mechanical property gradients, and contact friction on the evolution of wear damage using 304 stainless steel as a model material;
(3) To compare the wear damage characteristics of materials with a homogeneous microstructure with that of materials with a graded microstructure.

## 2. Materials and Methods

**Figure 1:**
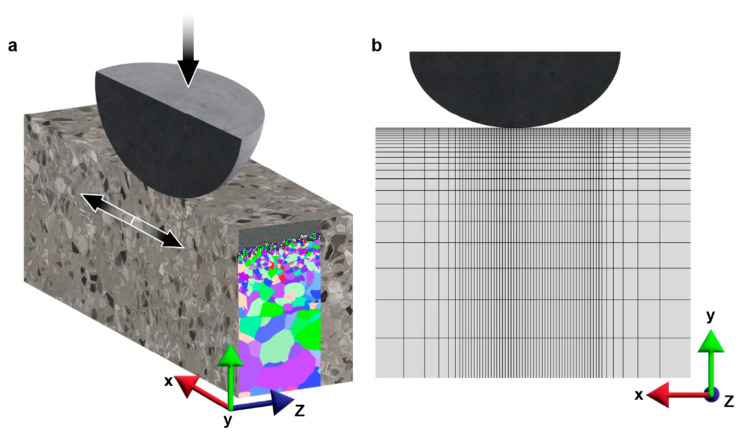
Schematic of simulation setup (**a**) and a close-up view of the finite-element mesh setup near the contact region (**b**).

In the present study, fretting damage is evaluated based on the extent of plastic deformation since significant plastic straining can result in surface damage and the nucleation of cracks [45,46]. To analyze this, finite element analysis (FEA) is conducted using the commercial software Abaqus (version 6.14, Dassault Systèmes, Waltham, MA, USA). The fretting simulation model is generated with a simplified geometry consisting of a rigid spherical indenter (with diameter, *d* = 1 mm) in contact with an elastic–plastic half-space. A schematic of the fretting model is depicted in Figure 1a. Due to the symmetry involved, only one-half of the half-space block needs to be modeled, which is 500 μm deep, 300 μm wide, and 100 μm thick. In this study, a hexahedral mesh with a biased mesh size was employed in three dimensions. Specifically, a mesh volume density of ∼2.6 μm^−3^ was utilized in the contact region (Figure 1b). The bias is introduced to the mesh to ensure a higher mesh density near the contact area, where plastic deformation is more prominent. The three-dimensional FEA fretting sliding simulations were performed under displacement-controlled conditions. The half-space is subjected to loading by the rigid half-sphere with a vertical load of 500 mN in the -*y* direction, while the sample slides along the *x* (and -*x*) direction a total of six times, with a 3 μm sliding distance in each step. The sliding direction is reversed in each subsequent step to simulate the fretting motion. The contact plane, set as the *x*-*z* plane, is the top surface of the half-space block.

**Figure 2:**
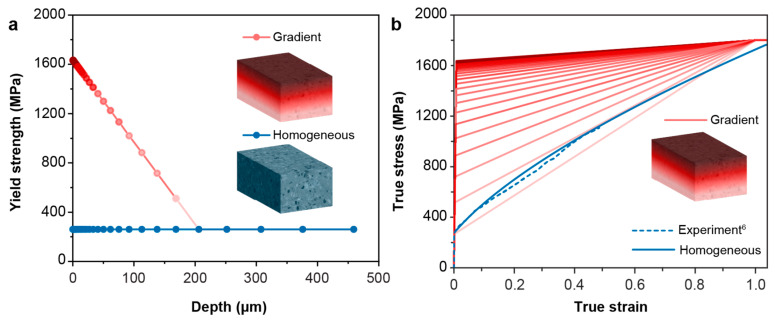
The constitutive properties for the homogenous and gradient models. (**a**) The yield strength distribution along the depth direction for the gradient structure and the homogeneous structure. (**b**) True stress–strain curves used in simulations for the gradient model corresponding to different yield strengths in each layer (solid red curves), the experimental curve for the homogeneous material (dotted blue curve), and the fitted stress–strain curve used in simulations for the homogeneous model.

The gradient microstructure is modeled as a fully bonded, multilayered structure consisting of 30 homogeneous layers with varying mechanical properties. As shown in Figure 2a, the input data for the yield strength and the elastic–plastic properties for each layer were constructed based on a previous study on SMAT-processed 304 stainless steels [6], fitted with a linearly varying yield strength through thickness until reaching the baseline value of the untreated material. When the deformation exceeds the yield strength, the plastic deformation of each layer in the gradient structure is modeled using a linear hardening behavior, with the change in its strain-hardening coefficient being proportional to the change in the linearly varying yield strength versus depth, before reaching a saturation value of 1800 MPa at 100% strain (Figure 2b). For homogeneous structures, the yield strength was the same as the as-received 304 stainless steels [6], and the stress (σ) versus equivalent plastic strain behavior (${\overset{-}{\varepsilon }}^{p}$) is fitted with a Swift model [47,48] (Figure 2b):

$$\sigma =1505{\left(0.06+{\overset{-}{\varepsilon }}^{p}\right)}^{n}$$

Here, *n* is the hardening exponent, which is varied from 0.25 to 0.85 in order to investigate the effect of strain hardening on the fretting behavior in the homogeneous structure. For both homogenous structures and gradient structures, a Young’s modulus of 200 GPa and Poisson’s ratio of 0.3 are used. The contact friction coefficient between the spherical indenter and the homogenous structure or the gradient structure is varied from 0.1 to 0.6 in order to investigate the effect of the friction coefficient on the fretting behavior of the substrate materials.

To ensure the accuracy and reliability of our simulation results, we have verified our simulation model through sensitivity analysis (Figures S1 and S2). Overall, the simulation setup and methodology employed in this study provide a robust and reliable framework for evaluating fretting damage based on plastic deformation.

We note that the formation of strain-induced martensite is a prevalent phenomenon in the plastic deformation of AISI 304 stainless steels. For instance, in the SMAT-treated sample, the martensite phase is formed at intersections of twins with sizes ranging from several nanometers to sub-micrometers [49]. This unique phase transformation process facilitates grain refinement procedures. Furthermore, the strain-induced transformation increases the hardening rate at smaller strains. But at larger strains, the transformation saturates, resulting in a sharp decrease in strain hardening [50]. As mentioned in prior research [51,52,53,54], the phase transformation process is a critical factor that reduces the corrosion resistance of 304 SS. However, in this study, we are primarily focusing on the yield strength and hardening behavior of individual layers, and microstructural changes such as grain-growth or phase transformation are not considered. We will take into account the effect of such microstructural changes in a future study.

## 3. Results

### 3.1. The Comparison between the Homogenous Structure and the Gradient Structure

**Figure 3:**
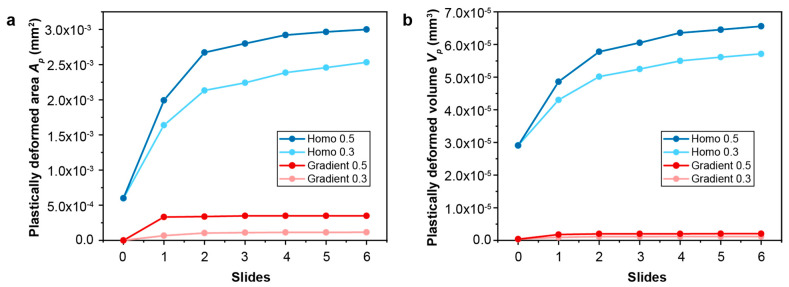
The comparison of fretting damage between gradient and homogeneous structures: (**a**) plastically deformed area and (**b**) plastically deformed volume at friction coefficients of 0.3 and 0.5.

**Figure 4:**
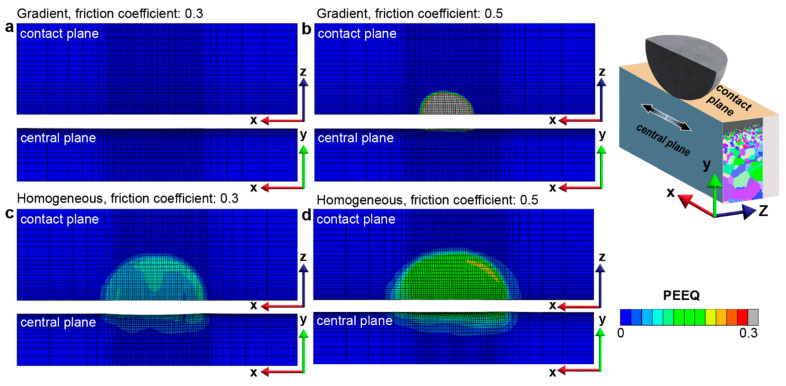
The comparison of equivalent plastic strain (${\overset{-}{\varepsilon }}^{p}$, or PEEQ) distribution between (**a**,**b**) gradient structures and (**c**,**d**) homogeneous structures after 6 sliding reversals. Here, (**a**–**d**) correspond to the cases with friction coefficients of 0.3 and 0.5, respectively. Due to the small PEEQ values in case (**a**), the plastic zone is not visible; however, it is clearly seen in Figure 5a,c using a color bar with a maximum value of 0.009.

The contact friction conditions used in the FEA models for predicting fretting damage on the homogeneous structure and the gradient structure are derived from experimental observations. A nanostructured surface can significantly reduce the steady-state friction when significant plastic deformation is involved during sliding [16]. Under high loads, the steady-state friction coefficient, *f*, with a coarse-grained (homogeneous) surface layer is ~0.35–0.55, while the *f* observed in the materials with gradient surface layers is ~0.3–0.53 [16,20]. The extent of fretting damage, which, in this study, is evaluated by the plastically deformed surface area $A_{p}$ and the plastically deformed volume $V_{p}$, is assessed for the homogenous structure and the gradient structure for two friction coefficients of 0.3 and 0.5, which, respectively, represent relatively low and relatively high friction conditions. In addition, the magnitude and distribution of the (accumulated) equivalent plastic strain, ${\overset{-}{\varepsilon }}^{p}\left(x,y,z\right)$, and the plastic strain tensor, $\varepsilon^{p}\left(x,y,z\right)$, are also used to characterize fretting-induced plastic deformation and to distinguish between *elastic shakedown* and *plastic shakedown* behaviors. As shown in Figure 3a,b, compared to the homogeneous structure, the gradient structure exhibits significantly lower fretting damage with a smaller plastically deformed area and volume during the six slides of frictional sliding. As the yield strengths of the surface layers are much higher in the case of graded material than in the case of homogeneous material, the graded material can support the same amount of load involving a much smaller plastically deformed volume. It is interesting to observe that the maximum equivalent plastic strain (${\overset{-}{\varepsilon }}^{p}$, or PEEQ) introduced in the contact region is much higher in the gradient structure when *f* = 0.5 but is much lower when *f* = 0.3 compared to the two cases of homogeneous structures (Figure 4).

**Figure 5:**
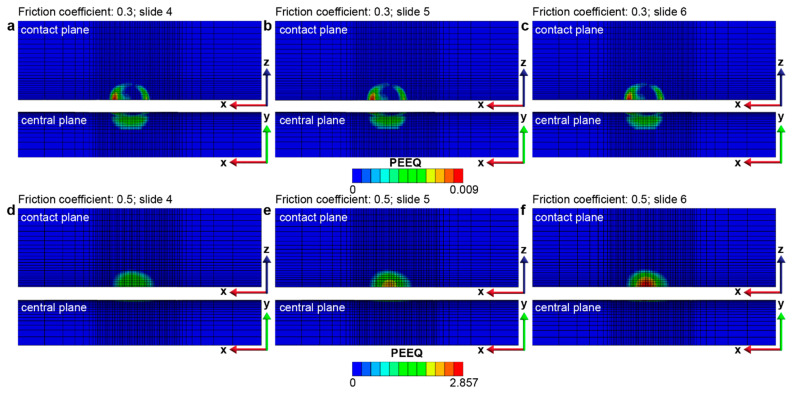
Equivalent plastic strain (PEEQ) distribution and shakedown behavior after 4, 5, and 6 sliding reversals in the gradient structure with a friction coefficient of 0.3 (**a**–**c**) and 0.5 (**d**–**f**), respectively. The results show that the gradient structure with a friction coefficient of 0.3 (**a**–**c**) displays *elastic shakedown* behavior, with a constant plastic strain distribution (i.e., $∆{\overset{-}{\varepsilon }}^{p}\left(x,y,z\right)$ = 0), while the gradient structure with a friction coefficient of 0.5 (**d**–**f**) exhibits *plastic shakedown* behavior with both the plastically deformed surface area and volume remaining constant after 4 sliding reversals, whereas PEEQ keeps increasing (i.e., $∆{\overset{-}{\varepsilon }}^{p}\left(x,y,z\right)$ > 0) within the plastically deformed volume.

**Figure 6:**
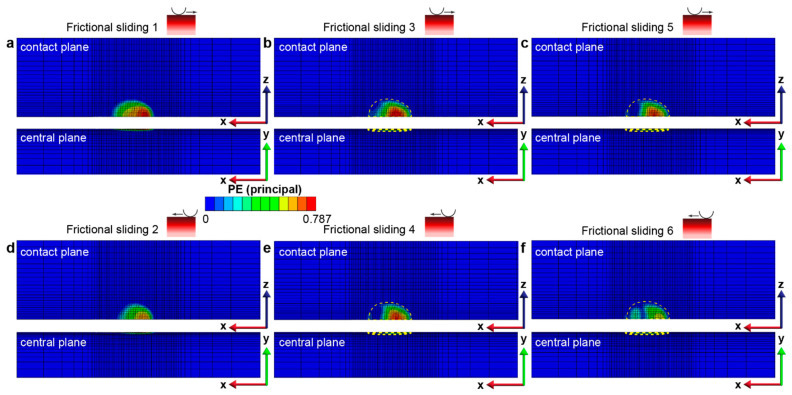
Maximum principal plastic strain distribution in the gradient structure after each of the 6 fretting sliding reversals: (**a**–**c**) correspond to forward sliding instances 1, 3, and 5, while (**d**–**f**) correspond to backward sliding instances 2, 4, and 6, respectively. The yellow dashed region denotes the plastically deformed volume where the (accumulated) equivalent plastic strain ${\overset{-}{\varepsilon }}^{p}\left(x,y,z\right)>0$.

Furthermore, the gradient structure with *f* = 0.3 manifests no discernible increment in the PEEQ magnitude (i.e., no additional plastic deformation) throughout the plastically deformed region, which corresponds to the *elastic shakedown* response (as depicted in Figure 5a–c). In contrast, the gradient structure with *f* = 0.5, while both its plastically deformed surface area and volume are kept constant after four sliding reversals (see Figure 4), shows an obvious increase in the PEEQ within the plastically deformed volume, indicating *plastic shakedown* behavior (as illustrated in Figure 5d–f and Figure 6). More details of the plastic shakedown behavior exhibited by the gradient structure with *f* = 0.5 are shown in Figure 6, where the distribution of the maximum principal plastic strain (taken from the strain tensor $\varepsilon^{p}\left(x,y,z\right)$) is kept within the plastically deformed volume established in previous sliding steps and decreases in peak value with an additional back-and-forth sliding cycle after the third sliding reversal.

### 3.2. Fretting Behavior of Gradient Nanostructured Materials

As shown in Section 3.1, the graded nanostructured materials exhibit superior fretting damage resistance when compared to homogeneous materials. In order to optimize the design of gradient structures for enhanced fretting damage resistance, the effects of contact friction and the gradient layer thickness on the plastic deformation characteristics subject to multiple fretting sliding reversals are investigated.

#### 3.2.1. The Effect of Contact Friction

**Figure 7:**
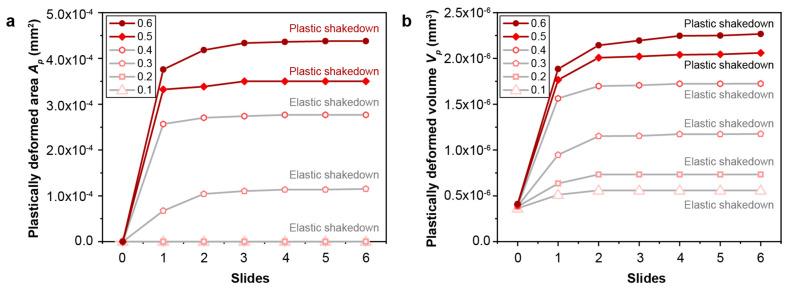
The comparison of the plastically deformed surface area (**a**) and volume (**b**) for gradient structures with different friction coefficients. The gradient structure with a friction coefficient ranging from 0.1 to 0.4 exhibits *elastic shakedown* behavior, while the gradient structure with a friction coefficient of 0.5–0.6 displays *plastic shakedown* behavior.

**Figure 8:**
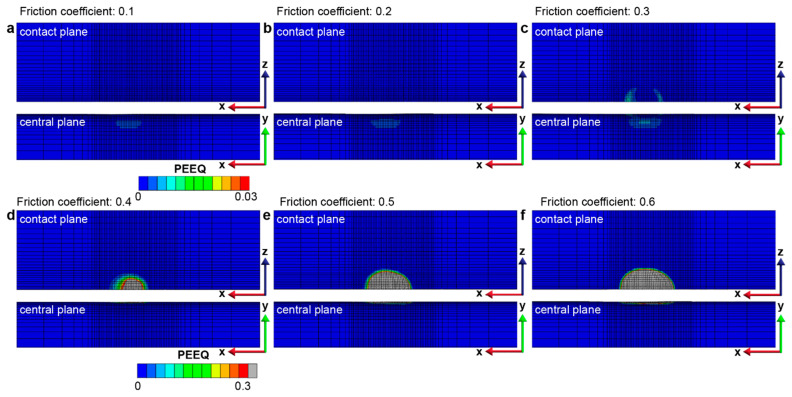
The equivalent plastic strain distribution in the gradient structure after 6 sliding reversals with the friction coefficient from (**a**–**f**) ranging from 0.1 to 0.6, respectively.

The effect of contact friction on the fretting response of gradient materials is examined by considering a range of friction coefficients from 0.1 to 0.6. As the contact friction between the indenter and the sample surface increases, the lateral shear force that develops in the contact region also increases, which results in an increase in the plastically deformed surface area and volume (Figure 7). For a particular fretting condition where the contact friction is a fixed constant, it is observed that the fretting-induced plastic deformation surface area and volume increase in the first two back-and-forth sliding cycles (up to four sliding reversals) but reaches a steady state after four sliding reversals (Figure 7). The onset of such a steady state in the plastically deformed surface area and volume with increasing sliding reversals is referred to as shakedown behavior [42]; when *f* = 0.1–0.4, *elastic shakedown* is observed, and when *f* = 0.5 and 0.6, *plastic shakedown* is achieved. From Figure 8, it can be seen that fretting under high friction coefficients (*f* = 0.3–0.6), which involves high tangential loads in the contact region, the plastic strain occurs on and near the contact surface with the maximum plastic strain at the surface (Figure 8c–e). However, for fretting under lower friction coefficients (*f* = 0.1 and 0.2), with lower tangential loads in the contact region, the plastically deformed volume is only observed in the subsurface regions of the gradient structure (Figure 8a,b). This is consistent with previous sliding contact analysis of an elastic homogenous half-space using a rigid circular sphere [32], which demonstrates that for lower friction cases (*f* ≤ 0.3), the first yield occurs beneath the contacting sphere, and it transitions towards the surface region with increasing friction. Moreover, using the same examination methods as shown in Figure 5 and Figure 6, at low friction coefficients (*f* = 0.1–0.4), the gradient structure displays an *elastic shakedown* response, while at high friction coefficients (*f* = 0.5 and 0.6), the gradient structure shows *plastic shakedown* behavior.

#### 3.2.2. The Effect of Gradient Layer Thickness

**Figure 9:**
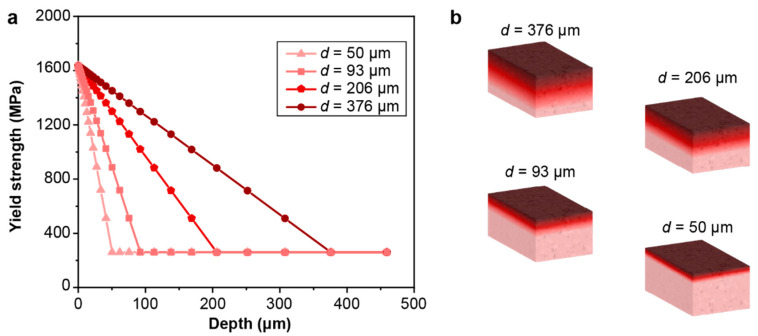
Modeling setup for structures with different gradient layer thicknesses. (**a**) The yield strength distribution versus depth for the gradient structure with different gradient layer thicknesses. (**b**) The schematic of structures with different gradient layer thicknesses.

**Figure 10:**
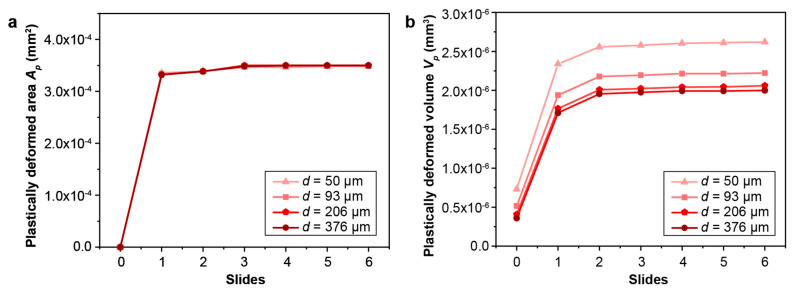
The comparison of (**a**) the plastically deformed area and (**b**) volume for gradient models with different gradient layer thicknesses with the friction coefficient *f* = 0.5. Plastic shakedown is achieved after 4 sliding reversals for these cases.

**Figure 11:**
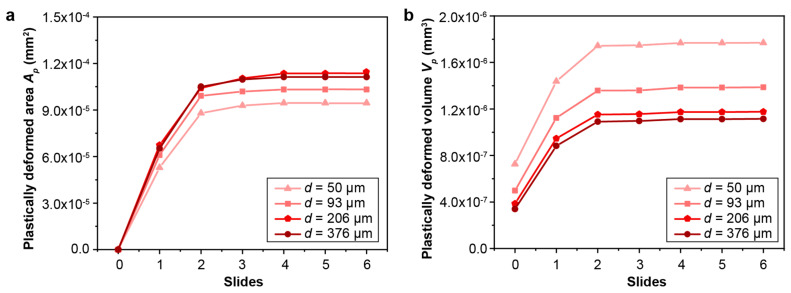
The comparison of the plastically deformed area (**a**) and volume (**b**) for gradient models with different gradient layer thicknesses with the friction coefficient *f* = 0.3. *Elastic shakedown* is reached after 4 sliding reversals for these cases.

**Figure 12:**
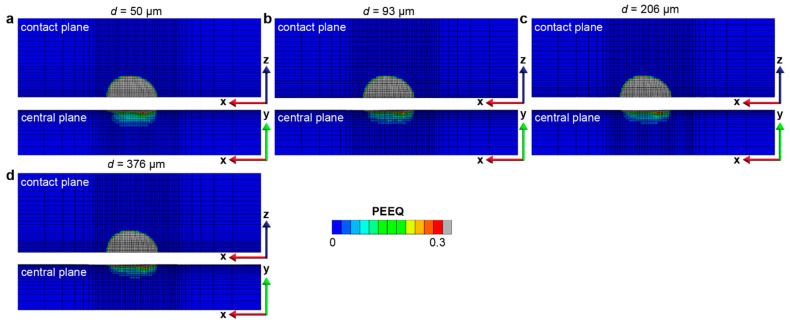
The equivalent plastic strain distribution in the gradient structure with different gradient layer thicknesses with the friction coefficient *f* = 0.5 after 6 sliding reversals. The gradient layer thicknesses for (**a**–**d**) are 50 μm, 93 μm, 206 μm, and 376 μm, respectively.

**Figure 13:**
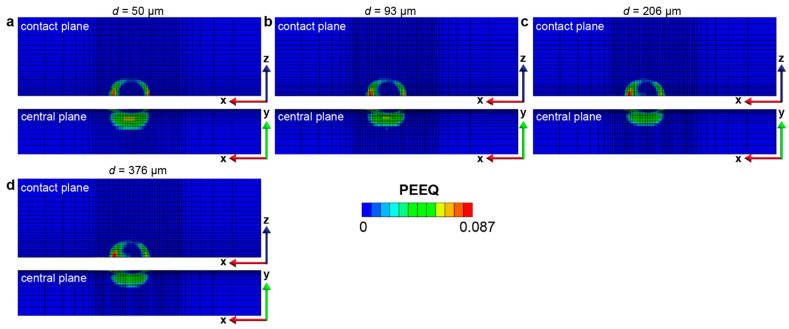
The equivalent plastic strain distribution in the gradient structure with different gradient layer thicknesses with the friction coefficient *f* = 0.3 after 6 sliding reversals. The gradient layer thicknesses for (**a**–**d**) are 50 μm, 93 μm, 206 μm, and 376 μm, respectively.

The effect of the gradient layer thickness on the fretting response of gradient materials is quantified by considering a range of gradient thicknesses (from 50 μm to 376 μm). The maximum and minimum plastic properties corresponding to the hard surface and soft core, respectively, remain the same. The same linear variations in the yield strength and hardening are assumed, as shown in Figure 2, except that the thickness of the gradient layer changes. Such a range of gradient thickness layers can be obtained by varying the surface treatment conditions, such as the SMAT process time and the SMAT process energy used (Figure 9). To assess the fretting behavior of the gradient materials, finite element simulations were conducted under conditions of relatively low and high contact friction (*f* = 0.3 and 0.5, respectively), as shown in Figure 10, Figure 11, Figure 12, Figure 13 and Figure S3. For both contact friction conditions (*f* = 0.3 and 0.5), the total plastically deformed volume of the material decreases with the increasing gradient thickness (Figure 10 and Figure 11). With a relatively high friction coefficient (*f* = 0.5), the plastically deformed surface area remains almost the same, while the plastically deformed volume decreases with an increasing gradient layer thickness (Figure 10 and Figure 12). *Plastic shakedown* is achieved after four sliding reversals. It is noted that the peak equivalent plastic strain increases with the increasing gradient layer thickness (Figure S3). On the other hand, with a relatively low friction coefficient (*f* = 0.3), the plastically deformed surface area slightly increases, while the plastically deformed volume decreases with an increasing gradient layer thickness (Figure 11 and Figure 13). *Elastic shakedown* is observed after four sliding reversals. In the cases with *f* = 0.3, the peak equivalent plastic strain increases slightly with the increasing gradient layer thickness (Figure 13). However, compared with values obtained in the corresponding cases with a lower friction coefficient (*f* = 0.3, Figure 11), both the plastically deformed surface area and volume calculated in the case with a higher friction coefficient (*f* = 0.5, Figure 10) are higher. In both friction conditions (*f* = 0.3 and 0.5), the maximum equivalent plastic strain (PEEQ) increases, while the plastically deformed volume decreases with the increasing gradient layer thickness.

The gradient nanostructured surface layer should be thick enough (hundreds of micrometers) to resist high cyclic friction and wear under high loads in experiments or practical applications. Otherwise, after a certain number of cycles, the gradient nanostructured layer may delaminate due to surface cracks and subsequent wear damage, resulting in a significant decrease in wear resistance [17].

### 3.3. Fretting Behavior of Homogeneous Materials

In order to fully understand the benefits of gradient structures for enhancing fretting damage resistance, the fretting response of the homogeneous baseline materials needs to be examined in more detail as well. Hence, the effects of contact friction and strain hardening of the homogeneous material on the fretting response are considered.

#### 3.3.1. The Effect of Contact Friction

**Figure 14:**
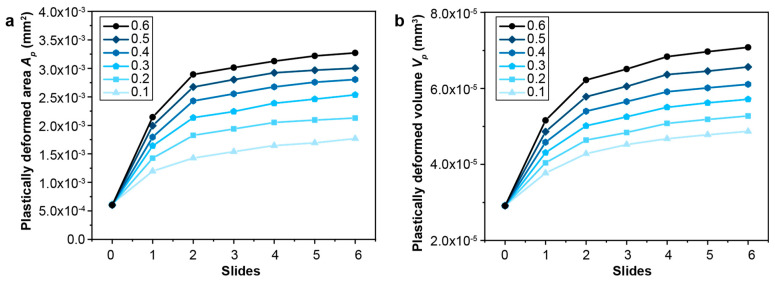
The comparison of the plastically deformed area (**a**) and volume (**b**) for the homogeneous structure with different friction coefficients.

**Figure 15:**
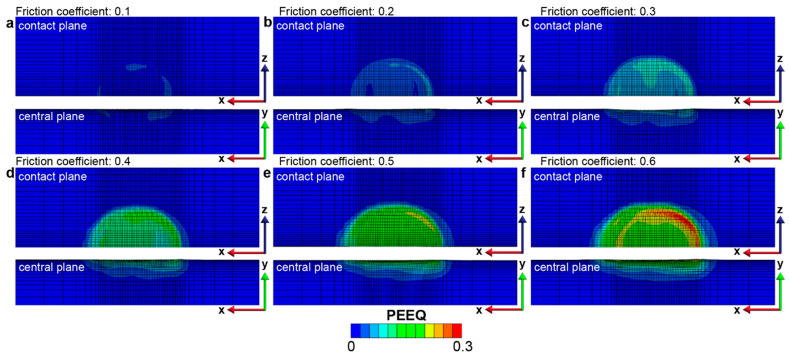
The equivalent plastic strain distribution in the homogeneous structure with different friction coefficients after 6 sliding reversals. The friction coefficients for the homogeneous cases (**a**–**f**) range from 0.1 to 0.6, respectively.

The effect of contact friction on the fretting response of homogeneous materials is assessed by considering a range of friction coefficients from 0.1 to 0.6. In general, the fretting-induced plastic deformation increases with an increase in contact friction (Figure 14). The maximum equivalent plastic strain occurs at the surface, and the magnitude of the plastic strain also increases with friction coefficients, as shown in Figure 15 and Figure S4. However, unlike in the case of gradient materials, elastic or plastic shakedown behavior is not observed during the six sliding reversals (i.e., three full back-and-forth fretting sliding cycles). The plastically deformed area and volume continue to increase with the increasing number of sliding reversals, even for a friction coefficient as low as 0.1.

#### 3.3.2. The Effect of Strain Hardening

**Figure 16:**
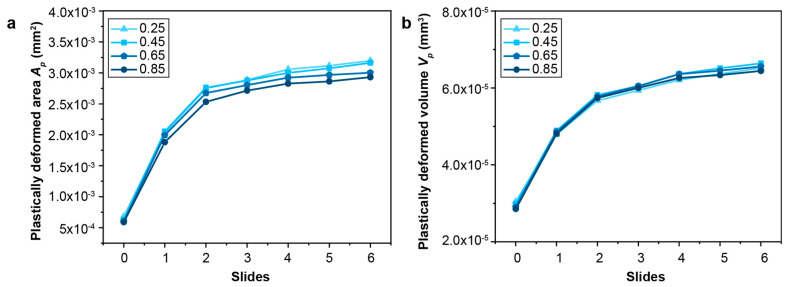
The comparison of (**a**) the plastically deformed area $A_{p}$ and (**b**) volume $V_{p}$ for homogeneous structures with different hardening exponents (0.25–0.85).

**Figure 17:**
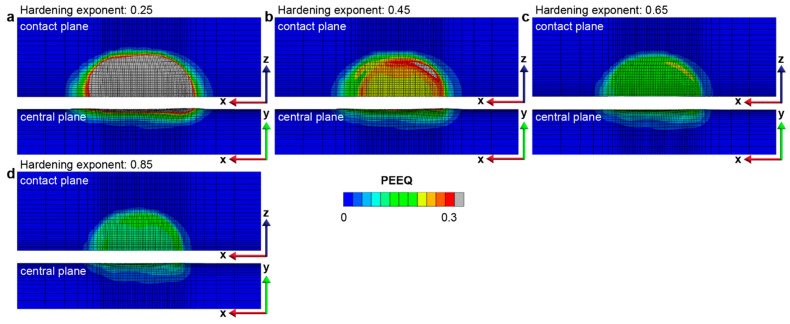
The equivalent plastic strain (PEEQ) distribution in the homogenous structure with different strain hardening exponents after 6 sliding reversals. Here, (**a**–**d**) represent the equivalent plastic distributions in the homogeneous structures with hardening exponent ranging from 0.25 to 0.85, respectively.

The effect of the strain-hardening characteristics of the homogeneous materials on their fretting sliding response is quantified by considering a range of strain-hardening exponents from 0.25 to 0.85, corresponding to those observed in low-strength stainless steels. It is observed that the frictional-sliding-induced plastically deformed volume is almost independent of the strain-hardening exponents. The fretting of materials with a relatively lower hardening exponent results in a relatively larger plastically deformed surface area but with a smaller depth, resulting in similar plastically deformed volumes for materials with different strain-hardening exponents (Figure 16 and Figure 17). The maximum equivalent plastic strain is much higher in the material with a lower strain-hardening exponent than that in the case of the material with a higher strain-hardening exponent (Figure 17 and Figure S5).

## 4. Discussion

The graded nanostructured materials exhibit a gradient in their microstructure from the nanograined region at the surface to the coarse-grained interior, with a corresponding gradient in the yield strength of the surface regions, which gradually decreases from the surface to the interior. Due to the higher yield strength of the nanograined surface layers, the gradient material resists plastic deformation, and therefore, the fretting-induced plastically deformed surface area and volume are smaller when compared to the coarse-grained homogeneous material. This observation is confirmed by the results of the FEA simulations, as shown in Figure 3 and Figure 4. The gradient nanostructured material is demonstrated to exhibit superior resistance to repeated sliding contact as compared to the coarse-grained materials.

Furthermore, the gradient material exhibits *elastic shakedown* behavior at lower friction coefficients (*f* = 0.1–0.4) with almost solely elastic deformation after reaching shakedown, and *plastic shakedown* behavior at higher friction conditions (*f* = 0.5–0.6) where a steady state in the fretting-induced plastically deformed volume is obtained (Figure 7) under repeated fretting sliding. However, such shakedown behavior is not observed under similar frictional sliding conditions for the homogeneous coarse-grained material.

For the gradient materials (and the homogenous materials with smaller friction coefficients of *f* = 0.1–0.3), the maximum von Mises stress, and hence the maximum equivalent plastic strain, occurs at the edge of the contact area during the repeated fretting sliding process (Figure 8 and Figure 15). This observation is similar to the plastic strain distribution observed in the fretting behavior in previous studies [41]. For the cases with higher friction coefficients (i.e., between 0.4 and 0.6), the maximum equivalent plastic strain location switches to the center of the contact region at the surface. In addition, due to the higher tangential load induced by higher friction coefficients, most of the contact region yields plastically, and the plastically deformed volume transforms into an elliptical shape. The observed evolution of the shape and extent of the fretting-induced plastically deformed volume is attributed to the interplay between the shear stresses and the normal stresses that are developed in the contact regions under different contact friction conditions [42].

For the gradient materials considered in the present study, the plastic deformation zone is typically confined to a shallow region immediately beneath the nanograined surface (Figure 8 and Figure 12). When the gradient layer thickness is small, the high contact-induced stresses may extend beneath the thin nanograined layer (which has high yield strength) to the homogeneous base material (which has much lower yield strength), and thus, more plastic deformation is produced under the contact surface. In the limiting case of a coarse-grained homogeneous material with a thin-film layer of a nanostructured surface, where the plastic strain difference between the nanostructured surface layer and the coarse-grained subsurface could be large, delamination or detachment of the nanostructured surface layer may also happen [55]. However, when the gradient layer thickness is large enough, e.g., 206 μm, as considered in this study, the gradient layers are more effective in shielding the underlying coarse-grained homogeneous material from high stresses, and the plastic deformation is largely confined to the nanostructured gradient layers which have higher yield strengths. On the other hand, the plastic properties of the homogeneous material, such as yield strength and the strain hardening exponent, influence the fretting sliding behavior as expected, with the materials which have a lower hardening exponent exhibiting a higher magnitude of the equivalent plastic strain (Figure 16 and Figure 17).

It is to be noted that in the experiments conducted on graded nanostructured metals, depending on the contact stress conditions, two plastic deformation mechanisms may be observed [12,13,14,24,56,57]: the dislocation activities in the entire plastically deformed region and mechanically driven grain size changes in the gradient region, which can significantly affect the mechanical properties of the gradient structure. For example, after a large number of frictional sliding passes, the nanostructured surface may coarsen to ultrafine grains during the fretting or sliding process, and the coarse-grained subsurface could be refined to smaller grain sizes due to severe plastic deformation [13,58]. These deformation mechanisms can help to accommodate large plastic strains and assist with suppressing shear localizations. With the limited number of sliding reversals simulated, however, no microstructural evolution is considered in this study. Nevertheless, deformation-induced microstructural changes during fretting sliding will be an important topic for investigation in future efforts.

## 5. Conclusions

Nanostructured graded materials have demonstrated exceptional mechanical properties, such as high hardness and fatigue strength. However, limited studies are available on the deformation characteristics of GNS materials under fretting sliding conditions. Therefore, this study focused on understanding the fundamental structure–property relations in GNS materials under fretting and frictional sliding conditions. In this study, 304 stainless steel was utilized as a model system to investigate the behavior of GNS materials. Moreover, the GNS materials are modeled as layered structures with a yield strength gradient. Most GNS metal materials, including copper [24,57,58], titanium [8], and alloys [9,15], due to their grain size gradient in the depth direction, possess a yield strength gradient from a high yield strength on the surface to a low yield strength in the interior region. The principal conclusions from the present study are as follows:

(1) A three-dimensional finite element model was developed to predict the fretting-induced plastic deformation characteristics and both elastic and plastic shakedown behaviors in graded materials.
(2) Graded nanomaterials with their lower plastically deformed contact volumes demonstrate superior resistance to repeated sliding contact as compared to coarse-grained materials.
(3) Contact friction has a significant effect on the fretting-induced plastic deformation characteristics in the graded nanomaterials and homogeneous materials. In general, the size of the plastically deformed zone increases with increasing friction.
(4) The thickness of the nanograded layer plays an important role in determining the frictional sliding resistance of the material. Materials with a thin gradient layer thickness may exhibit slightly smaller plastically deformed surface area but higher plastically deformed volume upon fretting sliding and could be susceptible to increased wear or delamination compared to materials with a sufficiently thick nanograined gradient layer.
(5) Graded nanomaterials have demonstrated *elastic shakedown* behavior with lower friction coefficients (*f* = 0.1–0.4) and *plastic shakedown* behavior with higher friction coefficients (*f* = 0.5–0.6), enhancing fretting resistance. In contrast, homogeneous coarse-grained materials under similar fretting sliding conditions do not exhibit such a shakedown behavior. *Elastic shakedown* behavior observed at relatively low friction in the gradient structure is more desirable than *plastic shakedown* behavior observed in the cases with relatively high friction because no additional plastic deformation accumulates with an increasing number of fretting sliding cycles in *elastic shakedown*.
(6) The fretting sliding response of a homogeneous material depends more on the contact friction than on the strain-hardening characteristics of the material, with larger plastically deformed zones expected under higher friction conditions.

The conclusions obtained in this study using 304 stainless steel as a model system are also expected to be broadly applicable to many GNS metallic materials and would be helpful for designing GNS materials for many applications such as high-load and high-speed automotive engines, industrial machinery, aerospace components, and nuclear reactors, where enhanced fretting and frictional sliding resistance are required.

## Data Availability

Not applicable.

## Supplementary Materials

The following supporting information can be downloaded at: https://www.mdpi.com/article/10.3390/nano13101584/s1, Figure S1: The comparison of fretting damage on the gradient model with different mesh density. Figure S2: Equivalent plastic strain (PEEQ) distribution 6 sliding reversals in the gradient structure with a friction coefficient of 0.5 at different mesh density (a–c). Figure S3: The equivalent plastic strain (PEEQ) distribution in the gradient structure with different gradient layer thicknesses with the friction coefficient *f* = 0.5 after 6 sliding reversals; Figure S4: The equivalent plastic strain distribution in the homogeneous structure with different friction coefficients after 6 sliding reversals. Figure S5: The equivalent plastic strain (PEEQ) distribution in the homogenous structure with different strain hardening exponents after 6 sliding reversals.
