# Supplementary material for: Modeling Fretting Wear Resistance and Shakedown of Metallic Materials with Graded Nanostructured Surfaces
Source: Nanomaterials (Basel). 2023 May 9;13(10):1584. doi: 10.3390/nano13101584 (PMC10222345; doi:10.3390/nano13101584)
Supplement: Supplementary file 1 [file nanomaterials-13-01584-s001.zip › nanomaterials-2316458-supplementary.pdf]

Supporting information

Fretting wear resistance and shakedown of metallic materials with graded nanostructured surfaces

Ting Yang [1], T. A. Venkatesh [2] and Ming Dao [1,*]

[1] Department of Materials Science and Engineering, Massachusetts Institute of Technology, Cambridge, MA 02139, USA; tingy@mit.edu
[2] Department of Materials Science and Chemical Engineering, Stony Brook University, Stony Brook, NY 11794, USA; t.venkatesh@stonybrook.edu
* Correspondence: mingdao@mit.edu

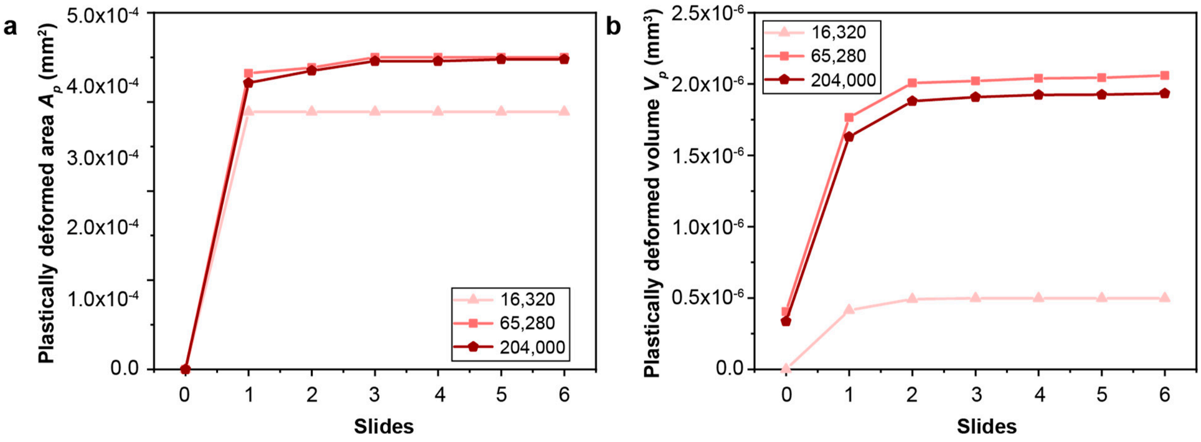

Figure S1| Comparison of fretting damage on the gradient model with varying mesh densities. Plastically deformed area **(a)** and plastically deformed volume **(b)** were computed using different total numbers of elements: 16,320, 65,280, and 204,000, respectively, while maintaining a constant friction coefficient of 0.5 across all three cases.

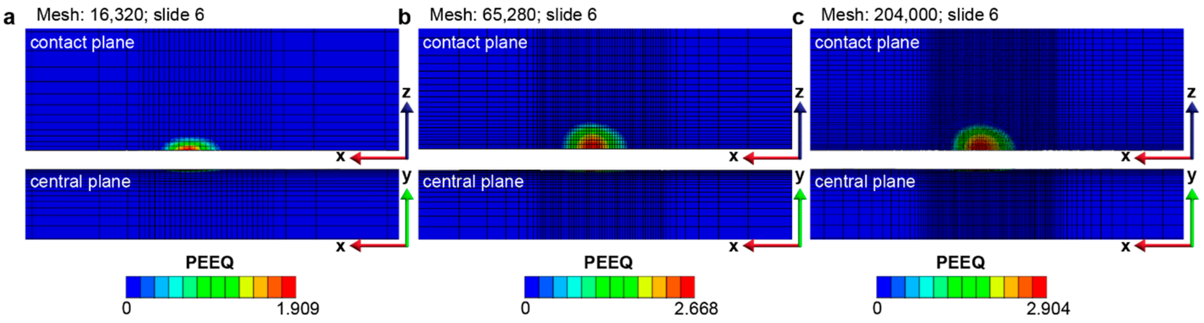

Figure S2| Equivalent plastic strain (PEEQ) distribution after 6 sliding reversals in the gradient structure with a friction coefficient of 0.5 with varying mesh densities. The total element numbers used are **(a)** 16320, **(b)** 65280, and **(c)** 204000, respectively.

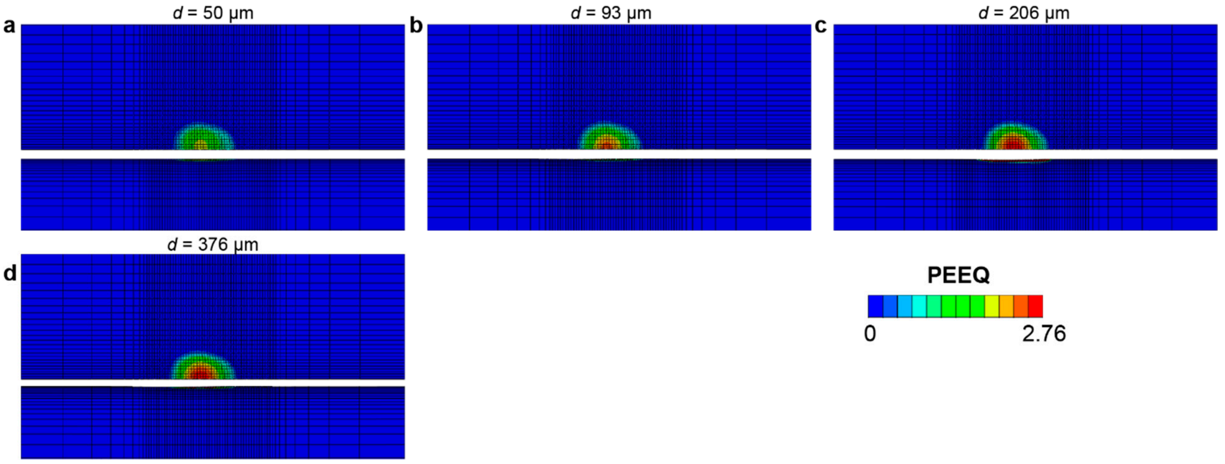

Figure S3| The equivalent plastic strain (PEEQ) distribution in the gradient structure with different gradient layer thicknesses with the friction coefficient *f* = 0.5 after 6 sliding reversals. The gradient layer thickness ranges from the lowest to the highest in **(a-d)**. Note that the color bar range is set differently than that used in Fig. 12, highlighting the regions with high PEEQ values.

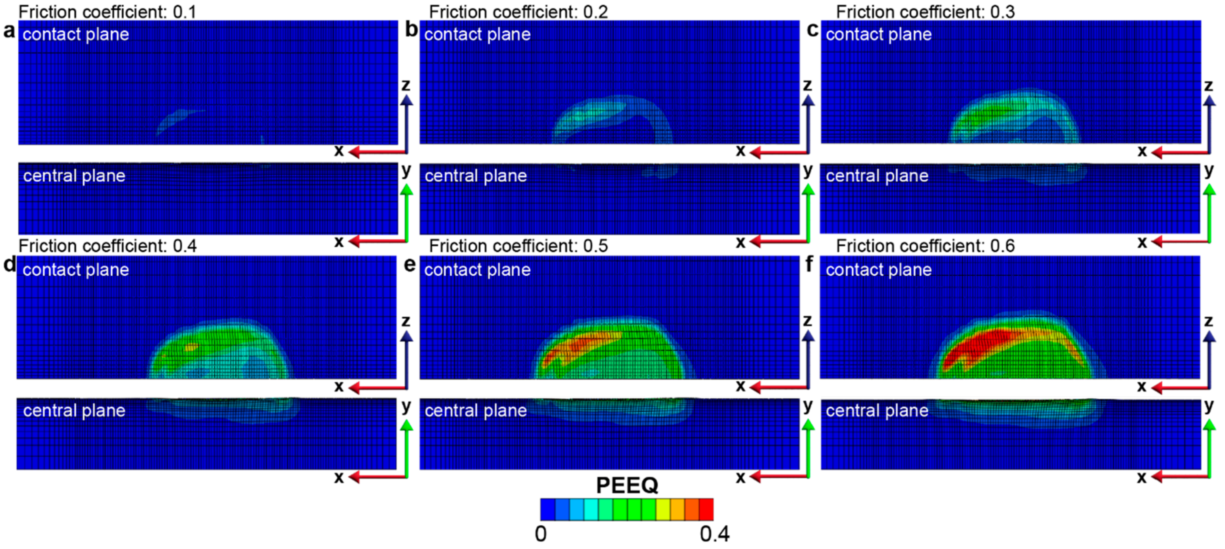

Figure S4| The equivalent plastic strain distribution in the homogeneous structure with different friction coefficients after 6 sliding reversals. The friction coefficient for the homogeneous cases in **(a-f)** ranges from 0.1 to 0.6, respectively. Note that the fine-mesh region is larger than that used in Fig. 15.

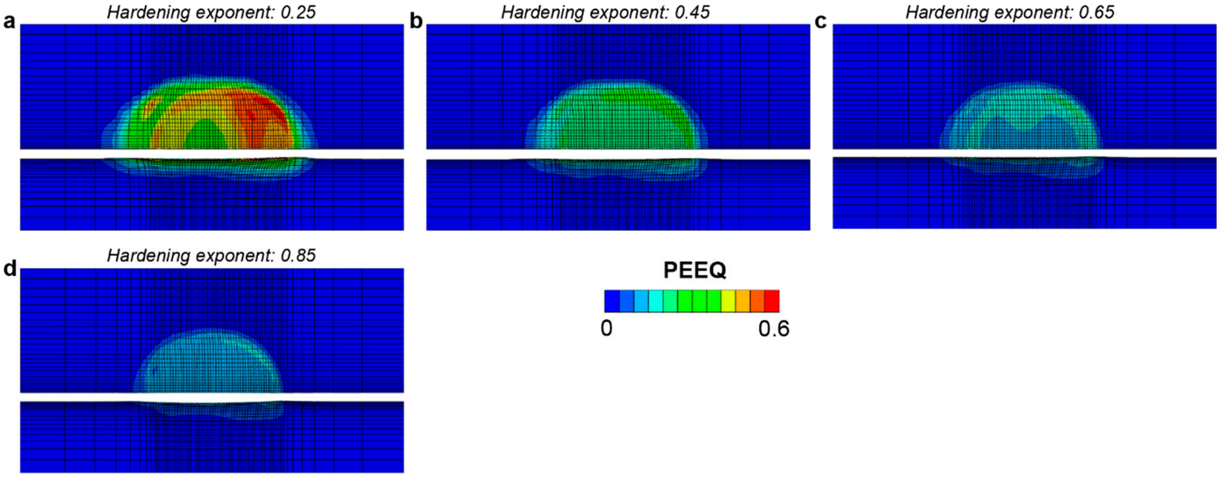

Figure S5| The equivalent plastic strain (PEEQ) distribution in the homogenous structure with different strain hardening exponents after 6 sliding reversals. Here contours in **(a-d)** represent the equivalent plastic distributions in the homogeneous structures with hardening exponent ranging from 0.25 to 0.85, respectively. Note that the color bar range is set differently than that used in Fig. 17, highlighting the regions with high PEEQ values.
